# Efficacy and safety of Pro Re Nata regimen without loading dose ranibizumab injections in retinal vein occlusion

**DOI:** 10.12669/pjms.313.7218

**Published:** 2015

**Authors:** Erkan Unsal, Kadir Eltutar, Pınar Sultan, Hulya Gungel

**Affiliations:** 1 Erkan Unsal, Istanbul Research and Training Hospital, Ophthalmology Department, Kasap Ilyas Mah Org Abdurrahman Nafiz, Gurman Cd, PK 34098, Fatih, Istanbul, Turkey; 2 Kadir Eltutar, Istanbul Research and Training Hospital, Ophthalmology Department, Kasap Ilyas Mah Org Abdurrahman Nafiz, Gurman Cd, PK 34098, Fatih, Istanbul, Turkey; 3 Pınar Sultan, Istanbul Research and Training Hospital, Ophthalmology Department, Kasap Ilyas Mah Org Abdurrahman Nafiz, Gurman Cd, PK 34098, Fatih, Istanbul, Turkey; 4 Hulya Gungel, Istanbul Research and Training Hospital, Ophthalmology Department, Kasap Ilyas Mah Org Abdurrahman Nafiz, Gurman Cd, PK 34098, Fatih, Istanbul, Turkey

**Keywords:** Macular thickness, Ranibizumab, Retinal Vein Occlusion

## Abstract

**Objectives:**

To evaluate the effects and safety of intravitreal ranibizumab on visual acuity and anatomic results in the treatment of macular edema due to retinal vein occlusion (RVO).

**Methods:**

Forty Six eyes of 45 patients who were administered intravitreal ranibizumab because of macular edema due to Retinal Vein Occlusion (RVO) were included in this retrospective clinical study. During monthly follow-up, the best corrected visual acuity values in terms of LogMAR with The Early Treatment Diabetic Retinopathy Study (ETDRS) chart, central macular thickness (CMT), and complications were examined. Cases were classified as central retinal vein occlusion (CRVO), superotemporal branch retinal vein occlusion (BRVO), and inferotemporal BRVO. We only included RVO patients but using ETDRS chart for the vision measurement.

**Results:**

In all follow-up months, there was a significant increase in BCVA in all RVO cases and in superotemporal BRVO cases after the first injection of ranibizumab. Although there was no significant increase in the 1^st^ month of follow-up period compared to pre-treatment, there was significant increase in 2-6 months in inferotemporal BRVO patients. There was no statistically significant increase in 1^st^ and 2^nd^ month follow-up periods compared to pre-treatment; however there was a significant increase in 3-6 months in the CRVO patients. There was a significant decrease in average CMT measurements in all follow-up months compared to pre-treatment in all RVO cases, in superotemporal and inferotemporal BRVO cases. There was no significant decrease in average CMT measurements in the 1^st^, 2nd, and 3^rd^ months compared to pre-treatment although there was a significant decrease in 4-6 months in cases included in the CRVO patients.

**Conclusions:**

Intraocular ranibizumab injections provided rapid, effective treatment for macular edema due to RVO with low rates of ocular and nonocular safety events. However, repeated injections and frequent follow-up intervals may be required.

## INTRODUCTION

Macular edema, a leading cause of vision loss, has been reported in 60% of cases of retinal vein occlusion (RVO).^1^ The use of grid laser photocoagulation is known to be an effective treatment option in treatment of macular edema due to branch retinal vein occlusion (BRVO).^2^ However, grid laser photocoagulation has limited application due to the risk of developing iatrogenic paracentral scotoma; in addition, some eyes are resistant to treatment.^3^ In addition grid laser photocoagulation treatment has not been recommended in macular edema due to CRVO.^4^

Ischemia that develops as a result of vascular occlusion causes the release of vascular endothelial growth factor (VEGF) from the retina and disruption of the blood retinal barrier.^5^ VEGF contribute to the development of macular edema. VEGF has proinflammatory properties and there are VEGF receptors on inflammatory cells.^6^

After understanding that VEGF has an important role in the pathogenesis of macular edema, ranibizumab has been used in treatment of macular edema due to RVO with intravitreal administration.^7^-^9^ The aim of treatment is to minimize photoreceptor damage by decreasing the duration of edema.^10^

The aim of this study was to evaluate the effects of intravitreal injection of 0.5 mg/0.05 ml ranibizumab (Lucentis, Genentech, Inc., South San Francisco, CA), a VEGF inhibitor, on visual acuity and anatomic results in treatment of macular edema due to RVO.

## METHODS

Medical records of 46 eyes of 45 patients who had macular edema due to CRVO, superotemporal and inferotemporal branch retinal vein occlusion (BRVO), who were injected with intravitreal ranibizumab were studied retrospectively between October 2011 and January 2014. Patients were followed up for at least 6 months in the Retinal Unit of Eye Diseases Clinic in Istanbul Education and Research Hospital.

The local ethics committee consent was obtained for this study. The study was conducted in accordance with the Declaration of Helsinki. Before administration of intravitreal lucentis injection, all patients were informed in detail about the side effects of the drug and its administration and their consent was taken.

Ophthalmoscopic examinations were performed on the patients before treatment. For the measurement of best corrected visual acuity (BCVA), ETDRS chart and logMAR (Logarithm of the minimum angle of resolution or recognition) scoring were used. Biomicroscopic examination and intraocular pressure (IOP) measurement was performed with Goldmann tonometry. The fundal examination was performed with a 90 D lens by dilating the pupils with 2.5% phenylephrine and 1% tropicamide. *Central foveal retinal thickness (CMT) (μm)* measurement was obtained with fundus photography, fundus fluorescein angiography (FA), and spectral domain optical coherence tomography (Optovue OKT (V 5.1, RTVue 100-2, Optovue, Fremont, CA, USA)).

Patients with Ischemic vein occlusion, iris/retina/disc neovascularization, vitreoretinal surgery or intravitreal bevacizumab/triamcinolone/Ozurdex injections were excluded from the study (Table-I).

**Table-I:** Eligibility Criteria for the study.

*Criteria of inclusion*
The presence of non-ischemic CRVO and BRVO
At least 6 months follow-up duration is required
Absence of iris / retina / disc neovascularization
Having BCVA level of at least light perception level
Adequate pupillary dilation and media transparency for OCT-FA
*Criteria of exclusion*
Ischemic BRVO and CRVO
The presence of iris / retina / disc neovascularization
Having intravitreal triamcinolone / bevacizumab / ozurdex implant injection
Vitreoretinal surgery history
Macular edema due to other reasons
Aphakia, the anterior chamber lens
Active inflammation, infection
Uncontrolled systemic diseases (DM,HT,SVO)
History of cataract surgery in the last 6 months
Laser photocoagulation application history

*Abbreviations:* CRVO, central retinal vein occlusion; BRVO, branch retinal vein occlusion; BCVA, best-corrected visual acuity; OCT, optical coherence tomography; FA, fluorescein angiographi; DM, Diabetes mellitus; HT, Hypertension.

Patients with an ischemic area larger than 5 disks in BRVO, and 10 disks in CRVO in FA were accepted as ischemic BRVO and ischemic CRVO. Ranibizumab injection was applied to patients with a diagnosis of RVO in FA and with CMT of greater than 250 microns in central section with OCT measurements.

After pupillary dilatation, the application was performed by a single vitreoretinal surgeon under sterile conditions in an operating room with the standardized procedure.^2^-^4^ After the injection optic nerve perfusion was checked. Lomefloxacin HCl (Okacin, Novartis) was prescribed four times per day for one week after the injection.

In accordance with the BRAVO and CRUISE studies, additional ranibizumab was injected for those with BCVA ≤ 20/40 according to Snellen chart or with CMT 250 ≥ μm measured by OCT. Patients were observed for injection-related complications after the application and they were checked monthly.

### Statistical analysis

The relationship between BCVA and CMT before injection and during follow-up was investigated using the paired t test and Wilcoxon paired samples t test with ‘SPSS 15.00 for Windows’ (SPSS Inc., Chicago, Illinois, USA) software. A p<0.05 was considered statistically significant.

## RESULTS

Forty six eyes from 45 patients, including 19 (42.2%) female and 26 (57.7%) male with an average age of 60.3±11.87 years were included in the study (Table-II). Before the initiation of treatment, the duration of retinal vein occlusion was found to be 3.76±0.97 months (range: 2-5 months) on average. During the average follow-up period of 8.35±5.6 (7-19) months, ranibizumab was injected once in 18 eyes, twice in 10 eyes, 3 times in 14 eyes, and 4 times in 4 eyes.

**Table-II:** Characteristics of patients.

Patient/eye number	45/46
Average age	60,3±11,87 (40-85)
Gender (F/M)	19(%42.2)/26(%57.7)
Right/left eye	20 (%43,5) / 26 (%56,5)
CRVO/Superotemporal BRVO/Inferotemporal BRVO	14 (%30,4) / 22 (% 47,8) /10 (%21,7)
Treatment starting time (month)	3.76±0.97 (2-5)
Average follow-up period (month)	8.35±5.6 (7-19)
Phakic / Pseudophacic	29/17
Number of Injection (mean± SD (min-max))	
RVO/CRVO/Superotemporal BRVO/Inferotemporal BRVO	2.08±1.02 (1-4) / 2.57±0.93 (1-4) / 2.0±0,87 (1-3)/1.60±1.26 (1-4)
Number of cases with diagnosis of Diabetes Mellitus	
RVO/CRVO/Superotemporal BRVO/Inferotemporal BRVO	8/4/3/1
Number of cases with diagnosis of glaucoma	
RVO/CRVO/Superotemporal BRVO/Inferotemporal BRVO	4/3/1/0
Number of cases with diagnosis of hypertension	
RVO/CRVO/Superotemporal BRVO/Inferotemporal BRVO	11/5/5/1

*Abbreviations:* RVO, retinal vein occlusion; CRVO, central retinal vein occlusion; BRVO, branch retinal vein occlusion; SD, standard deviation; M, Male; F, Female.

The average number of injection per eye was 2.08±1.02 (1-4) in all cases, 2.57±0.93 (1-4) in CRVO cases, 2.0±0.87 (1-3) in superotemporal BRVO, and 1.60 ± 1.26 (1-4) in inferotemporal BRVO (Table-II). 22 (68.7%) BRVO cases (n=32) were superotemporal BRVO, and 10 (31.3%) of them were inferotemporal BRVO (Table-II).

### Efficacy

**Fig.1:**
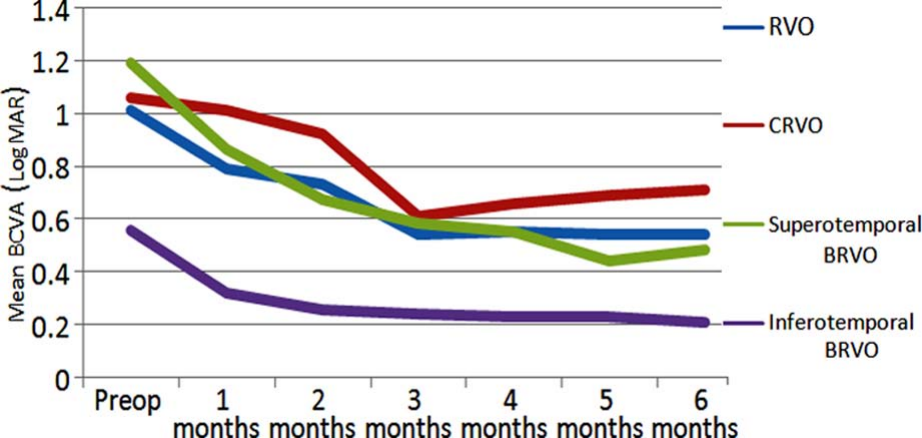
Mean BCVA (LogMAR) before and after treatment. Abbreviations: BCVA, best-corrected visual acuity; LogMAR; logarithm of the minimum angle of resolution; RVO, retinal vein occlusion; CRVO, central retinal vein occlusion; BRVO, branch retinal vein occlusion

There was a significant increase in average BCVA in RVO cases in all follow-up months compared to the pre-treatment period (p<0.05, Paired t test). While there was no significant increase at the 1^st^ and 2^nd^ months compared to the pre-treatment period (p>0.05, Wilcoxon test), there was a significant increase in the 3-6^th^ month phase in CRVO cases (p<0.05, Wilcoxon test). In superotemporal BRVO cases, there was a significant increase in all follow-up periods (p<0.05, Wilcoxon test). While there was no significant increase in the 1^st^ month compared to the pre-treatment period (p>0.05 Wilcoxon test), there was a significant increase in the 2-6 month follow-up periods in the inferotemporal BRVO cases (p<0.05, Wilcoxon test) (Table-III) (Fig.1).

**Table-III:** Comparison of average BCVA and CMT at the beginning and follow-up months in RVO, CRVO, superotemporal BRVO, inferiorotemporal BRVO groups.

	Parameter	Initial	1 months	2. months	3. months	4.months	5. months	6. months
RVO (n=46) p^1^	BCVA (LogMAR)	1.01±0.49	0.79±0.43	0.73±0.46	0.54±0.37	0.55±0.41	0.54±0.36	0.54±0.36
	Mean±SD, Range	(0.1-1.8)	(0.15-1.51) (p=0.001)	(0.15-1.8) (p=0.001)	(0.00-0.130) (p=0.000)	(0.00-0.130) (p=0.000)	(0.00-0.130) (p=0.000)	(0.0-1.30) (p=0.000)
	Central foveal retinal thickness (μm), Mean±SD, Range,	503.6±118 (276-713)	430.7±130 (248-678) (p=0.000)	449±175 (233-811) (p=0.021)	343±123 (214-725) (p=0.000)	354±132 (225-731) (p=0.000)	353±137 (241-711) (p=0.000)	364±130 (206-719) (p=0.000)
CRVO (n=14) P^2^	BCVA(LogMAR)	1.06±0.42	1.01±0.28	0.92±0.53	0.61±0.51	0.66±0.43	0.69±0.49	0.71±0.44
	Mean±SD, Range,	(0.5-1.8)	(0.7-1.51) (p=0.673)	(0.15-1.80) (p=0.063)	(0.00-1.30) (p=0.012)	(0.00-1.30) (p=0.018)	(0.00-1.30) (p=0.020)	(0.00-1.30) (p=0.022)
	Central foveal retinal thickness (μm) Mean±SD, Range	569±118 (401-713)	541±97 (400-678) (p=0.139)	592±156 (423-811) (p=0.814)	421±186 (236-725) (p=0.072)	448±156 (216-730) (p=0.042)	424±163 (219-728) (p=0.024)	438±167 (206-719) (p=0.004)
Superotemporal BRVO (n=22) P^2^	BCVA(LogMAR)	1.19±0,47	0.86±0.41	0.67±0.38	0.58±0.30	0.55±0.29	0.44±0.30	0.48±0.29
	Mean±SD, Range	(0.4-1.8)	(0.15-1.51) (p=0.004)	(0.15-1.30) (p=0.008)	(0.00-1.00) (p=0.000)	(0.00-1.00) (p=0.000)	(0.00-1.00) (p=0.000)	(0.00-1.00) (p=0.000)
	Central foveal retinal thickness (μm) Mean±SD, Range	495±106 (331-682)	406±121 (273-652) (p=0.017)	366±130 (233-627) (p=0.008)	316±72 (214-437) (p=0.000)	322±88 (209-446) (p=0.000)	339±97 (204-476) (p=0.000)	331±101 (215-548) (p=0.000)
İnferotemporal BRVO (n=10) P^2^	BCVA(LogMAR)	0.56±0.39	0.32±0.25	0.26±0.19	0.24±0.17	0.23±0.16	0.23±0.15	0.21±0.18
	Mean±SD, Range	(0.1-1.0)	(0.15-0.80) (p=0.057)	(0.1-0.63) (0.021)	(0.1-0.63) (p=0.022)	(0.1-0.63) (p=0.018)	(0.1-0.63) (p=0.021)	(0.1-0.63) (p=0.012)
	Central foveal retinal thickness (μm) Mean±SD, Range	429±102 (278-559)	328±70 (248-412) (p=0.005)	321±55 (243-387) (p=0.018)	306±49 (247-389) (p=0.012)	312±54 (251-339) (p=0.011)	318±59 (244-397) (p=0.010)	312±49 (263-391) (p=0,005)

*Abbreviations:* BCVA, best-corrected visual acuity; LogMAR; logarithm of the minimum angle of resolution; RVO, retinal vein occlusion; CRVO, central retinal vein occlusion; BRVO, branch retinal vein occlusion; SD, standard deviation;

1: paired t test;

2: Wilcoxon signed rank test. (<0.05 indicates statistical significance).

Visual acuity at the end of the six month follow-up showed that there was a 2 level or more increase in 30 (65.2%) of 46 eyes, there was less than a 2 level increase in 2 eyes (4.3%), there was more than a 2 level decrease in 3 eyes (6.5%), and there was no change in 11 eyes (23.9%), (Table-IV).

**Table-IV:** Visual acuity changes in 6th month follow-up.

BCVA	The number of eye
≥2 level increase	30
<2 level increase	2
no change	11
>2 level decrease	3

Abbreviations: BCVA, best-corrected visual acuity.

### Macular thickness

**Fig.2:**
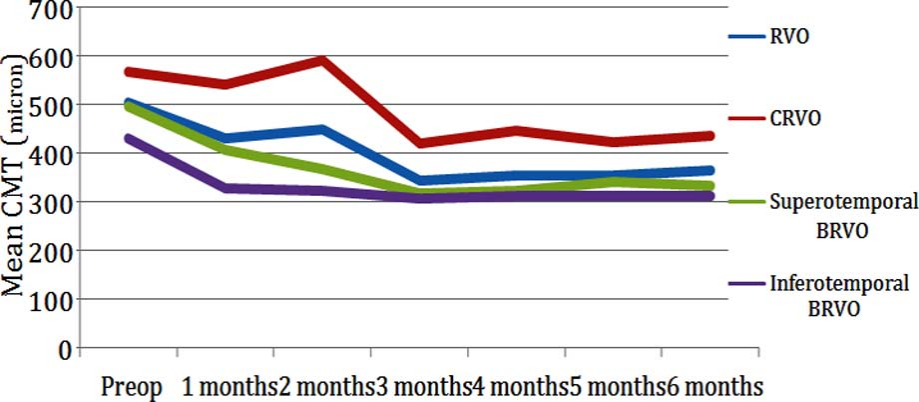
Mean CMT before and after treatment. Abbreviations: CMT, Central foveal retinal thickness; RVO, retinal vein occlusion; CRVO, central retinal vein occlusion; BRVO, branch retinal vein occlusion.

In RVO cases, there was a significant decrease in average CMT in all follow-up months compared to the pre-treatment period (p<0.05, Paired t test). In subgroup cases with CRVO (n=14), there was no significant decrease in the 1^st^, 2^nd^, and 3^rd^ months compared to pre-treatment (p>0.05, Wilcoxon test), but there was a significant decrease from 4-6^th^ months (p<0.05, Wilcoxon test). In superotemporal BRVO and inferotemporal BRVO cases, there was a significant decrease in all follow-up periods (p<0.05, Wilcoxon test) (Table-III) (Fig.2).

In RVO, CRVO, superotemporal and inferotemporal BRVO groups, the maximum reduction in CMT thickness after the first injection occurred in the 3^rd^ month. CMT measurements were 343, 421, 214, and 306 μm respectively.

### Safety

There was no injection related complications such as endophthalmitis, retinal detachment, IOP increase or systemic side effects due to ranibizumab in patients except subconjunctival hemorrhage observed in 8 of 96 injections (8.3%).

IOP was obtained at 16.35±2.43 mmHg at the beginning, 16.5±2.03 mmHg at the 1^st^ month, 16.44±2.11 mmHg at the 2^nd^ month, 16.4±2.24 mmHg at the 3^rd^ month, 16.32±2.01 mmHg at the 4^th^ month, 16.04±1.98 mmHg at the 5^th^ month, and 16.52±2.64 mmHg at the 6^th^ month. There was no significant difference in IOP of check-up periods compared to the beginning (p>0.05, Paired t test). There was no ischemic type progression and no vitreous hemorrhage in any patients in this study.

## DISCUSSION

Retinal vein occlusion (RVO) is the second most common retinal vascular disease and frequently causes decreased visual acuity.^2^ It is known that the incidence of retinal vein occlusion increases with increasing age. The average age reported in the literature has been ranging from 48 to 69.5 years.^11^ In our study, the average age of 60.3±11.87 (40-85) was consistent with literature.

The superotemporal quadrant is known to be the most frequent involved quadrant in BRVO.^12^ Our study also supports this information since there was a superotemporal quadrant involvement in 68.7% of our patients. Macular edema is the most important reason of visual loss due to RVO.^13^ Many factors play a role in the pathogenesis of macular edema due to RVO.^9^

Although there are many medical and surgical treatment options for retinal vein occlusion, laser photocoagulation is still an important treatment.^2^,^14^ However, there may be complications with this treatment and grid laser photocoagulation is not applied today in CRVO.^4^,^15^

After understanding the role of VEGF in angiogenesis anti-VEGF drugs has been used in the treatment of ocular disease mediated by VEGF such as retinal vein occlusion. Ranibizumab, humanized mouse monoclonal fragment produced recombinantly, blocks all isoforms of VEGF A and degradation products.^16^

One-year results of the efficacy of 0.5 mg ranibizumab in macular edema, secondary to BRVO (BRAVO study)^17^ and CRVO (CRUISE study)^18^ were published. Later improvements obtained in the first 6 months continued with an average of 2.7 additional injections by BRAVO^17^ and with average of 3.6 additional injections by CRUISE.^18^ In the HORIZON study BRVO patients were administered 1-3 repeated injections and CRVO patients were administered 1-6 repeated injections.^19^ While the BRVO group maintained the visual gain, there was a reduction in visual acuity in the CRVO group. An increase in the CRVO group was remarkable in CMT measurements. Researchers attributed this case to reduced injection frequency. CMT measurement less than 250µ was detected in 75% of BRVO patients and in 56.9% of CRVO patients. In our study, CMT thickness was detected as less than 250 µ in 21.8% of BRVO patients and 14.2% of CRVO patients at the end of 6 months. CMT recovery rates may be low in our study because of the short period of follow-up and few injections. Loading with injection in the first 6 months and then continuing injections may improve anatomical recovery in the long term

Spaide et al. injected 0.5 mg intravitreal ranibizumab monthly 3 times to patients with CRVO.^20^ Before ranibizumab treatment, 16 of 20 cases were injected with bevacizumab or triamcinolone. After 12 months follow-up, there was an increase in visual acuity with an average of 8.5 injections in all cases. The most important contribution of our study to this study compared to BRAVO and HORIZON was having no injection before intravitreal ranibizumab or having no recovery laser for BRVO group. In our study, there was a significant increase in visual acuity with an average of 2.08 injections.

Kinge et al. published safety and efficacy of 0.5 mg ranibizumab in patients with macular edema due to CRVO. At the end of six months; there was a 12 letter increase in BCVA level and 304μm reduction in CMT.^21^ In our study, there was more than a 3 lines (more than 15 letters) increase in average visual acuity and average reduction of 131 μm in CMT in CRVO cases at the end of 6 months. The reason for having a lower than average CMT decrease in our study may be due to injecting no loading dose in the first 3 months and expecting an increase in macular thickness for repeated injections.

In the study of Campochiaro et al., intravitreal ranibizumab was injected to RVO patients and followed for 4 years. It was reported that resulting BCVA was 20/40 or better in approximately 80% of patients. At the end of the study, it was determined that ranibizumab treatment preserves visual potential in the long term in patients with macular edema due to RVO.^22^ In our study, 20/40 or more BCVA was 31% in the BRVO group and 28.5% in the CRVO group at an average follow-up period of 8.35 months. A lower rate of visual acuity was attributed to the short duration of follow-up period.

Kim et al.^23^ stated that multiple intravitreal anti-VEGF injections are not an important risk factor for IOP increase. There was no statistically significant increase in IOP in our study. The most common side effects observed in the study of HORIZON were retinal hemorrhages and conjunctival hemorrhages. Endophthalmitis was observed in 2 patients in the CRVO group. Sixteen patients indicated thromboembolic events. A total of 11 deaths were reported during the study.^19^

In our study, the most common side effect was subconjunctival hemorrhage (8.3%). During the follow-up period, none of the patients demonstrated ocular or systemic side effects due to ranibizumab or the injection. There was no mortality.

The weakness of our study is its retrospective nature and lack of determination of a fixed last check period for all cases. Positive aspects of our study are monthly follow-ups; classification of RVO patients into subgroups and isolating ranibizumab treatment effect by excluding patients administered laser photocoagulation and other injection drugs.

## CONCLUSION

Despite the promising results of intravitreal ranibizumab injection in the early period in macular edema due to retinal vein occlusion, there are still some questions with this treatment. There is a need for long term studies in order to present continuity of anatomical and functional recovery. It is necessary to investigate the relationship of visual acuity increase with age, sex, time to start treatment, and initial visual acuity in larger patient groups. Although ranibizumab is an effective treatment option in the treatment of macular edema due to retinal vein occlusion, its duration of effect may be extended by methods such as drug release systems since it has a short duration of effect.
